# Post-discharge follow-up of pediatric COVID-19 patients: insights into serological dynamics

**DOI:** 10.3389/fmicb.2024.1427327

**Published:** 2024-07-09

**Authors:** Shima Mahmoudi, Babak Pourakbari, Mohammad Ali Shahbabaie, Maryam Sotoudeh, Erfaneh Jafari, Reihaneh Hosseinpour Sadeghi, Setareh Mamishi

**Affiliations:** ^1^Biotechnology Centre, Silesian University of Technology, Gliwice, Poland; ^2^Pediatric Infectious Disease Research Center, Tehran University of Medical Sciences, Tehran, Iran; ^3^Department of Infectious Diseases, Pediatrics Center of Excellence, Children’s Medical Center, Tehran University of Medical Sciences, Tehran, Iran; ^4^Molecular Pathology and Cytogenetics Division, Pathology Department, Children’s Medical Center, Tehran University of Medical Sciences, Tehran, Iran

**Keywords:** COVID-19, SARS-CoV-2, antibody, seropositivity, children

## Abstract

**Introduction:**

Limited data are available regarding SARS-CoV-2 serological response dynamics in pediatric patients with COVID-19, contributing to gaps in our understanding of the immune response in this population. This study aimed to investigate SARS-CoV-2 IgG seropositivity in patients diagnosed with COVID-19 during hospitalization and 2–4 weeks after discharge.

**Methods:**

A cohort of patients, consisting of 31 individuals with confirmed acute COVID-19 infection and 27 diagnosed with Multisystem Inflammatory Syndrome in Children (MIS-C), was enrolled in the study. Follow-up clinic appointments were scheduled for 2–4 weeks post-discharge. During admission and follow-up, blood samples were collected from each patient for laboratory analysis. Anti-nucleoprotein SARS-CoV-2 IgG levels were determined using the Enzyme-Linked Immunosorbent Assay (ELISA) method.

**Results:**

In this study, a cohort of 58 patients was examined. At admission, 52% (*n* = 14) of MIS-C patients and 10% (*n* = 3) of acute COVID-19 patients had positive SARS-CoV-2 IgG test. Only 48 cases were referred to the hospital, and follow-up data was available for 20 cases with MIS-C and 28 cases with acute COVID-19. All patients (*n* = 15) who initially tested positive for SARS-CoV-2 IgG at admission remained positive serology during follow-up (100%). Among the 33 patients who initially tested negative, 12 (37.5%) showed a positive serology result during follow-up, while 21 (62.5%) remained negative. Within this subgroup, 11 cases (44%) were diagnosed with acute COVID-19, and one patient (12.5%) presented with MIS-C. Fourteen cases with acute COVID-19 infection (56%) and seven cases with MIS-C (87.5%) consistently showed negative serology results throughout the study. During follow-up, the median lymphocyte count demonstrated a significant difference, with 0.96 × 10^9^ cells per L (IQR: 0.75–3.0 × 10^9^ cells per L) in the SARS-CoV-2 IgG-negative group and 2.9 × 10^9^ cells per L (IQR = 1.33–7.22 × 10^9^ cells per L) in the SARS-CoV-2 IgG-positive group (*p*-value = 0.03). Patients who demonstrated seropositivity during the follow-up were associated with a notably severe disease (*p*-value = 0.028).

**Conclusion:**

Our study highlights the dynamic nature of SARS-CoV-2 IgG antibody responses in pediatric patients with COVID-19 infection. We observed a notable increase in seropositivity rates during follow-up. Furthermore, patients who were seropositive at follow-up demonstrated a severe disease course and lower lymphocyte counts compared to those with persistently negative serology. Our findings underscore the importance of longitudinal serological monitoring in understanding disease progression and immune response dynamics in pediatric COVID-19 cases.

## Introduction

In the early stages of the Coronavirus disease 2019 (COVID-19) pandemic, the proportion of confirmed cases among children was relatively low, and children were thought to be rarely affected by SARS-CoV-2 (Mahmoudi et al., 2020; Wu and McGoogan, 2020; Mohammadpour et al., 2022). Subsequent studies have consistently shown that children and adolescents are susceptible to SARS-CoV-2 infection, however, a large percentage of children are either asymptomatic or mildly symptomatic, and the true incidence of infection is underestimated due to low testing rates in children (Mahmoudi et al., 2023). Despite generally milder symptoms, children with COVID-19 are shown to be at a lower risk of hospitalization and severe complications (Vosoughi et al., 2022; Sedik, 2023). However, children with comorbidities may manifest more severe symptoms (Ekbatani et al., 2021; Mahmoudi et al., 2021). Moreover, in the course of the COVID-19 pandemic, a distinct phenomenon known as a multisystem inflammatory syndrome in children (MIS-C) has emerged, showing similarities to Kawasaki Disease (Mamishi et al., 2020, 2022a,b; Sancho-Shimizu et al., 2021). Both MIS-C and Kawasaki Disease present with mucocutaneous involvement, conjunctivitis, lymphadenopathy, and elevated inflammatory markers. However, the differentiation between MIS-C and Kawasaki Disease remains a topic of ongoing debate among medical professionals (Rostami-Maskopaee et al., 2022).

To complement the diagnosis, tests such as real-time reverse transcription PCR (RT-PCR) for SARS-CoV-2 and serology have been used. Serology relies on the detection of antibodies, specifically IgM and IgG, to offer valuable insights into an individual’s exposure to the SARS-CoV-2 virus. IgM antibodies, emerging early post-infection, act as initial indicators but exhibit a swift decline, limiting their reliability in later stages. On the contrary, IgG antibodies, appearing later in the infection timeline, persist for an extended period, offering a more stable marker of past infection or vaccination (Lippi et al., 2023). Limited data are available regarding SARS-CoV-2 serological response dynamics in pediatric patients with COVID-19, and there are substantial gaps in our understanding of the immune response in children (Bohn et al., 2023). A recent meta-analysis estimated the combined prevalence of positive SARS-CoV-2 IgM and IgG antibody tests in MIS-C to be 39% and 81%, respectively (Ghazizadeh Esslami et al., 2023). However, there is a scarcity of reports in pediatrics, particularly those employing a prospective or longitudinal design regarding SARS-CoV-2 serological response dynamics. This could be attributed to the challenges associated with accessing patient data and acquiring blood samples (Hoste et al., 2023).

This study aimed to investigate SARS-CoV-2 IgG seropositivity in patients diagnosed with COVID-19 during hospitalization and 2–4 weeks after discharge. This is a valuable approach to understanding the dynamics of antibody response over time, which can provide insights into the duration and persistence of immunity following infection.

## Materials and methods

### Study cohort

In this study, patients referred to Children’s Medical Center between September 2020 and April 2021, age ≤ 18 with a confirmed diagnosis of acute COVID-19 or MIS-C were enrolled. The sampling protocol was approved by the Ethical Committee of Tehran University of Medical Sciences, Tehran, Iran (IR.TUMS.CHMC.REC1399.069), and written consent was obtained from parents/guardians of patients before data collection.

Confirmation of acute COVID-19 infection relied on clinical symptoms (fever, cough, shortness of breath, fatigue, etc.), along with SARS-COV-2 positive RT-PCR (Mamishi et al., 2022b). MIS-C diagnosis was established based on a history of close contact with SARS-CoV-2, persistent fever (>38°C for more than 24 h), involvement of at least two organs including cardiac, renal, respiratory, hematologic, dermatologic, gastrointestinal or neurological, and laboratory results confirming systemic inflammation (Mamishi et al., 2020).

Patients who met the following criteria were eligible for discharge: they had been afebrile for more than 3 days, showed improved respiratory symptoms, had pulmonary imaging indicating significant resolution of inflammation, and/or tested negative for SARS-CoV-2 RT-PCR twice consecutively (sampling interval ≥ 24 h). After taking blood samples from the patients to perform laboratory and serology tests, the parents were asked to refer to the specialized clinic 2–4 weeks after discharge for follow-up. Moreover, the characteristics data including age, sex, presence of underlying diseases, immunodeficiency, and previous known COVID-19 contact were collected via a questionnaire. A complete blood count (White blood cell (WBC), neutrophil, lymphocyte, and platelet count), D-Dimer, ferritin, fibrinogen, erythrocyte sedimentation rate (ESR), C-reactive protein (CRP), and CD4^+^ and CD8^+^ T cell measurements were performed at admission and during a 2- to 4-week follow-up period after discharge. The cells were examined using a BD FACS Canto II flow cytometer (BD Biosciences). Based on the patients’ age, the lymphocyte subset percentage was divided into three categories: below normal range, within normal range, and above normal range (Mahmoudi et al., 2021; Shearer et al., 2003).

### Antibody detection

Serum samples were collected at admission and 2–4 weeks after discharge. For serological analysis, enzyme-linked immunoabsorbent assay (ELISA) was done on the serum samples using Pishtaz Teb Anti-nucleoprotein SARS-CoV-2 ELISA (IgG) and according to manufacturer’s instructions to measure antibody titer against SARS-CoV-2. The cut-off value was obtained according to the following formula: Cut-off value = 0.15 + the average light absorbance of the negative control (Cut-off Index (COI) = OD of sample/cut-off value). Values higher than 1.1 were considered positive and values lower than 0.9 were considered negative.

### Statistical analysis

The data was analyzed using SPSS software version 22. Categorical variables were reported as frequencies and percentages, whereas continuous variables were described as median and interquartile range (IQR) values. Chi-square tests were performed to compare variables, and two-sided *p*-values less than 0.05 were considered statistically significant.

## Results

In this study, a cohort of 58 patients were examined, with 31 individuals confirmed to have acute COVID-19 infection and 27 diagnosed with MIS-C.

**FIGURE 1:**
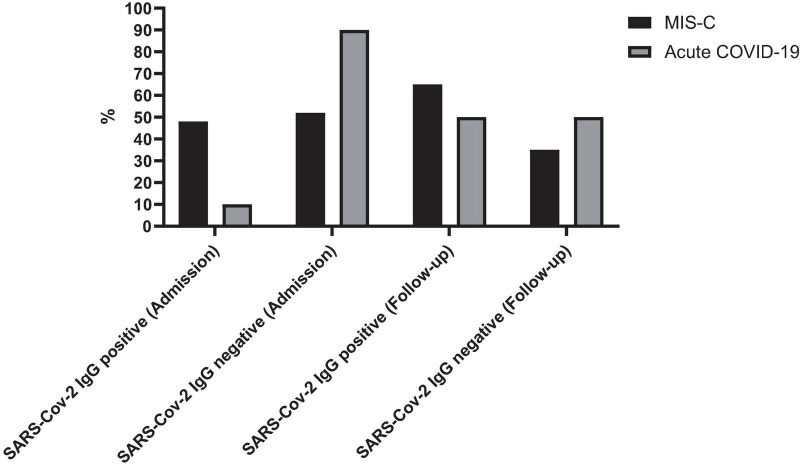
Seropositivity rates of SARS-CoV-2 IgG in patients with MIS-C and acute COVID-19 at admission and during follow-up. The figure illustrates the percentage of patients testing positive for SARS-CoV-2 IgG antibodies at admission and during follow-up.

The sex distribution was 55% males (*n* = 17) in the acute COVID-19 group and 56% males (*n* = 15) in the MIS-C group. Participants ranged in age from less than 1 month to 15 years, with a mean age of 71.3 ± 60.0 months. All cases were supposed to have follow-up laboratory tests and SARS-CoV-2 IgG tests 2–4 weeks later. Only 48 cases were referred to the hospital, and follow-up data was available for 20 cases with MIS-C and 28 cases with acute COVID-19. The mean time interval between the two tests was 20.7 ± 11.1 days for patients with MIS-C and 20.3 ± 11.2 days for patients with acute COVID-19. At admission, 52% (*n* = 14) of MIS-C patients and 10% (*n* = 3) of acute COVID-19 patients were SARS-CoV-2 IgG positive. During follow-up, the positive SARS-CoV-2 IgG was found in 56.3% (*n* = 27) of cases with MIS-C and 52% (*n* = 14) in cases with acute COVID-19 (Figure 1).

The mean age of patients with positive SARS-CoV-2 IgG did not significantly differ from those with negative SARS-CoV-2 IgG at admission (82 ± 56 months vs. 66.8 ± 61.4 months) (*p*-value = 0.39). A notable association was observed between the presence of underlying disease and the positivity of IgG at admission (*p*-value = 0.016). Among cases with negative SARS-CoV-2 IgG at admission, 46% (19 out of 41) had underlying disease, whereas only 12% of cases (2 out of 17) with positive SARS-CoV-2 IgG had underlying disease. Immune deficiency did not show a significant association with IgG positivity either at admission (*p*-value = 0.41) or during follow-up (*p*-value = 0.71). Conversely, the severity of the disease, classified as mild, moderate, or severe, was significantly associated with IgG positivity at admission. Remarkably, this association persisted and remained significant during follow-up (*p*-value = 0.008).

The examination of CD4^+^ and CD8^+^ T cell markers’ normality or impairment upon admission and subsequent follow-up revealed significant trends. In MIS-C patients, 60% (three out of five cases) with subnormal CD4^+^ T cell levels at admission normalized during follow-up. Conversely, among patients with acute COVID-19, only 20% (*n* = 2) with elevated CD4^+^ T cell levels experienced normalization during follow-up, while 70% (*n* = 7) maintained elevated levels at follow-up.

Regarding CD8^+^ T cell markers, there was an increase during follow-up in 71% of MIS-C cases (5 out of 7) and 27% (3 cases) of patients with acute COVID-19. Notably, half of the patients (*n* = 3) with elevated CD8^+^ T cell levels at admission demonstrated normal levels during follow-up (*p*-value = 0.026).

**FIGURE 2:**
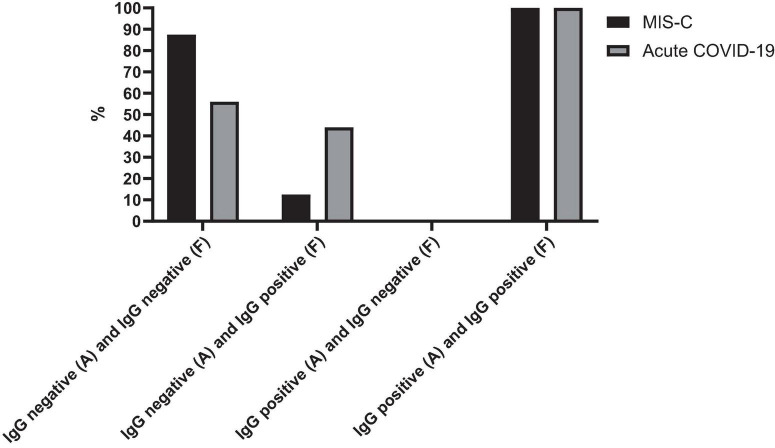
SARS-CoV-2 IgG test changes over time in cases with MIS-C and acute COVID-19. The figure presents the serology outcomes of 48 patients who underwent both admission and follow-up SARS-CoV-2 IgG testing. A, admission; F, follow-up.

**FIGURE 3:**
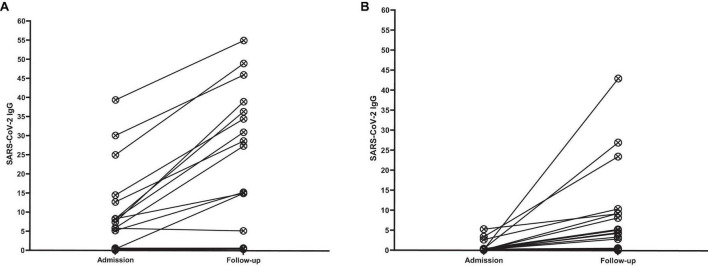
Anti-nucleoprotein SARS-CoV-2 IgG level in patients at admission and follow-up in panel **(A)** MIS-C group, **(B)** acute COVID-19 group. SARS-CoV-2 IgG was calculated based on the cut-off index (COI). Among the 8 patients with MIS-C who had a negative SARS-CoV-2 IgG level at admission, 7 (87.5%) remained negative during the follow-up, while 1 (12.5%) became positive in the follow-up. Among patients with acute COVID-19, 3 patients had a positive SARS-CoV-2 IgG level at both admission and follow-up. Among the 25 patients who had a negative SARS-CoV-2 IgG level at admission, 14 (56%) remained negative, while 11 (44%) showed a positive SARS-CoV-2 IgG level at follow-up.

**FIGURE 4:**
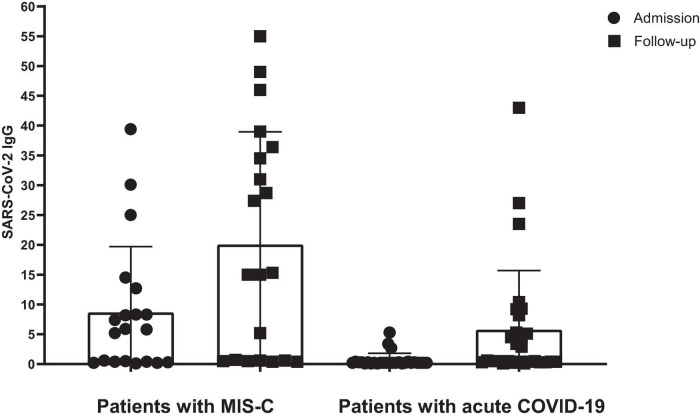
The level of SARS-CoV-2 IgG in patients with MIS-C and acute COVID-19. SARS-CoV-2 IgG was calculated based on the cut-off index (COI). Lines represent medians and interquartile ranges.

Figure 2 presents the serology results of 48 patients who underwent SARS-CoV-2 IgG testing both at the time of admission and during a follow-up period 2–4 weeks later. All patients (*n* = 15) who initially tested positive for SARS-CoV-2 IgG at admission remained positive serology during follow-up (100%). Among the 33 patients who initially tested negative, 12 (37.5%) showed a positive serology result during follow-up, while 21 (62.5%) remained negative (*p*-value < 0.001). Within this subgroup, 11 cases (44%) were diagnosed with acute COVID-19, and one patient (12.5%) presented with MIS-C. Fourteen cases with acute COVID-19 infection (56%) and seven cases with MIS-C (87.5%) consistently showed negative serology results throughout the study. Figure 3 shows the anti-nucleoprotein SARS-CoV-2 IgG levels in patients at admission and follow-up in patients with MIS-C and acute COVID-19. The median SARS-CoV-2 IgG COI level in patients with acute COVID-19 did not significantly differ between admission (0.2 [IQR: 0.2-0.3]) and follow-up (0.5 [IQR: 0.4–7.47]). On the other hand, patients with MIS-C exhibited a markedly higher median SARS-CoV-2 IgG COI level during follow-up (15.1 [IQR: 0.6–35.9]) compared to admission (5.85 [IQR: 0.4–11.6]) (*p*-value < 0.001) (Figure 4). Due to the limited sample size in the MIS-C group, which comprised only 8 patients, the examination of factors influencing seropositivity was exclusively conducted within the cohort diagnosed with acute COVID-19 infection. Table 1 provides a comparison of data for patients who initially tested negative for SARS-CoV-2 IgG antibodies at admission. It categorizes them based on subsequent negative or positive SARS-CoV-2 IgG test results during follow-up. The median WBC count was 5.16 × 10^9^ cells per L (IQR = 3.77–9.3 × 10^9^ cells per L) in the group that remained negative for SARS-CoV-2 IgG and 9.47 × 10^9^ cells per L (IQR = 3.88–11.47 × 10^9^ cells per L) in the subset that converted to SARS-CoV-2 IgG-positive status at follow-up, showing no significant difference (*p*-value = 0.2). During follow-up, the median lymphocyte count demonstrated a significant difference, with 0.96 × 10^9^ cells per L (IQR: 0.75–3.0 × 10^9^ cells per L) in the SARS-CoV-2 IgG-negative group and 2.9 × 10^9^ cells per L (IQR = 1.33-7.22 × 10^9^ cells per L) in the SARS-CoV-2 IgG-positive group (*p*-value = 0.03). Other parameters, including neutrophil count, platelet count, ferritin level, fibrinogen level, D-dimer level, CRP level, ESR, and CD4^+^ and CD8^+^ T cell percentage, did not exhibit significant differences between the two groups.

**TABLE 1:** Comparison of laboratory findings and clinical characteristics of pediatric patients with acute COVID-19, initially negative for SARS-CoV-2 IgG antibodies, stratified by subsequent SARS-CoV-2 IgG test results in follow-up.

Variable		IgG negative (A) and IgG negative (F) (*n* = 14)		IgG negative (A) and IgG positive (F) (*n* = 11)	
		*n*	%	*n*	%
Sex, male		9	64	6	54.5
Presence of underlying disease		7	50	8	73
Presence of immunodeficiency		4	29	3	27
Known COVID-19 contact		12	86	9	82
Severity of disease	Mild	9	64	2	18
	Moderate	3	21	2	18
	Severe	2	14	7	64
Laboratory findings					
WBC (×10^9^ cells per L)	Median (IQR)	5.16 (3.77–9.30)		9.47 (3.88–11.47)	
Lymphocyte (×10^9^ cells per L)	Median (IQR)	0.96 (0.75–3.0)		2.9 (1.33–7.22)	
Neutrophil (×10^9^ cells per L)	Median (IQR)	2.36 (1.2–5.17)		2.72 (1.22–8.97)	
Platelet (×10^9^ cells per L)	Median (IQR)	250 (168–381)		231 (185–236)	
Ferritin (ng/mL)	Median (IQR)	99 (50–359.5)		110 (88–200)	
Fibrinogen (mg/dL)	Median (IQR)	323 (234.2–460.5)		317 (279.2–413.2)	
D-Dimer (pg/mL)	Median (IQR)	0.6 (0.37–0.85)		0.35 (0.24–0.66)	
CRP (mg/L)	Median (IQR)	5 (3–13)		2.5 (1–14)	
ESR (mm/h)	Median (IQR)	10 (6–29.5)		17 (6–25)	
CD4^+^ T cell (%)	Median (IQR)	52 (47.5–55)		50 (40–56)	
CD8^+^ T cell (%)	Median (IQR)	32 (21–34.5)		25 (22–36)	

A, admission; F, follow-up; WBC, White Blood Cell; CRP, C-Reactive Protein; ESR, Erythrocyte Sedimentation Rate; IQR, Interquartile Range.

Among patients who tested negative for SARS-CoV-2 IgG at admission, 40.5% (15 out of 37) had CD4^+^ T cell counts higher than the normal range, 54% (20 out of 37) had counts within the normal range, and 5 (2 out of 37) had counts lower than the normal range. In comparison, among cases with positive SARS-CoV-2 IgG at admission, 33% (4 out of 12), 58% (7 out of 12), and 8% (1 out of 12) had lower, within the normal range, and higher than normal range CD4+ T cell counts, respectively (*p-*value = 0.013). Conversely, there was no significant difference observed in seropositivity concerning CD8^+^ T cells (*p*-value = 0.955).

**FIGURE 5:**
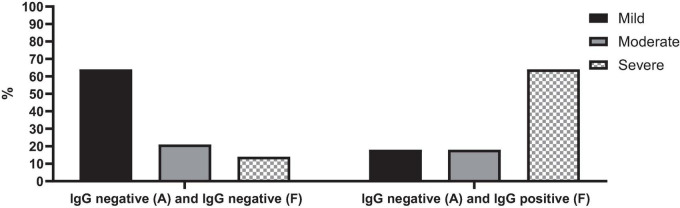
SARS-CoV-2 IgG serology test changes over time in cases with acute COVID-19 based on the severity of the disease.

Patients who were seropositive during the follow-up were associated with a notably severe disease (*p*-value = 0.028) (Figure 5). Specifically, among cases of acute COVID-19 with negative SARS-CoV-2 IgG tests upon admission but positive results during follow-up, 64% (*n* = 7) experienced severe COVID-19. In contrast, only 14% (*n* = 2) of cases with negative SARS-CoV-2 IgG tests both at admission and follow-up had severe COVID-19.

## Discussion

Our study contributes valuable insights into the serological response dynamics of pediatric patients diagnosed with acute COVID-19 and MIS-C. We observed a higher proportion of MIS-C patients testing positive for SARS-CoV-2 IgG at admission compared to acute COVID-19 patients, with 52% of MIS-C patients and only 10% of acute COVID-19 patients exhibiting seropositivity. Moreover, our longitudinal analysis revealed interesting trends in seropositivity rates during follow-up. All patients who initially tested positive for SARS-CoV-2 IgG at admission maintained positive serology throughout the follow-up period, indicating stable immune responses over time. In contrast, among patients initially testing negative, 37.5% exhibited seropositivity during follow-up. This observation underscores the dynamic nature of immune responses in pediatric COVID-19 cases and highlights the importance of longitudinal serological monitoring to capture changes in serostatus over time. This finding aligns with previous research indicating that antibody responses may take time to reach detectable levels, underscoring the importance of continued monitoring (Mamishi et al., 2021; Oygar et al., 2021).

In the previous study investigating the long-term antibody response to SARS-CoV-2 in children, detectable levels of anti-spike IgG and anti-nucleocapsid IgA/IgG/IgM antibodies were found for up to 270 days after the onset of symptoms (Dunay et al., 2023). Additionally, in longitudinal monitoring of antibody responses in pediatric populations, 95.8% of individuals maintained positive antibody status for a duration of up to 9 months post-infection (Oygar et al., 2021). In the study of Luo et al. (2020), during follow-up, SARS-CoV-2 IgG antibody was positive in 178 cases (10.7%), which was much lower than our report. In the study of Isoldi et al. (2021), an increase in SARS-CoV-2 IgG level was recorded in all patients after 1 month (mean IgG level 84.9 ± 24.7, median = 89.4, IQR = 32.3). In our study, patients who exhibited positive SARS-CoV-2 IgG during follow-up were associated with more severe disease manifestations and lower lymphocyte counts. This suggests a potential link between the strength of the immune response, disease severity, and the likelihood of seropositivity.

In adult patients, it is common to observe complete blood count abnormalities, especially lymphopenia, which has not been confirmed in children (Cohen et al., 2020; Rezaei et al., 2021). In our study, the median lymphocyte count in the SARS-CoV-2 IgG-negative group was 2.9 × 10^9^ cells per L (IQR: 1.33–7.22 × 10^9^ cells per L), while in the group with SARS-CoV-2 IgG-positive, it was 0.96 × 10^9^ cells per L (IQR: 0.75–3 × 10^9^ cells per L). In simpler terms, individuals with acute COVID-19 infection who were seropositive during the follow-up period had a lower lymphocyte count compared to those who remained negative serology.

All patients who tested positive for SARS-Cov-2 IgG at admission consistently maintained positive serology during follow-up (15 cases, 100%). In contrast, among the cohort of 33 patients who were negative for SARS-Cov-2 IgG at admission, 12 cases demonstrated seropositivity during follow-up (37.5%), while the remaining 21 patients remained seronegative (62.5%) (*p*-value < 0.001).

In a study by Zinszer et al. (2023) conducted during the early Omicron-dominant period from May 2022 to October 2022, a significant increase in seroconversion rates among seronegative children was observed. Specifically, children in this study were approximately 9–12 times more likely to seroconvert during this period compared to previous rounds of data collection conducted from May to September 2021 and November 2021 to February 2022 (Zinszer et al., 2023).

Several immunological hypotheses have been proposed to elucidate why children may exhibit lower rates of seroconversion (Selva et al., 2021). Firstly, differences in antibody profiles, including antibody isotypes and subclasses, along with variations in memory B-cell populations, may contribute to this phenomenon. Secondly, disparities in T-cell responses have been noted between SARS-CoV-2-infected children and adults. However, these findings are predominantly associated with disease severity (Toh et al., 2022).

SARS-CoV2 antibody responses have been observed to correlate with several factors, including age, viral load, sex, comorbidities (such as diabetes, cancer, and immunosuppression), as well as the severity of the disease (Toh et al., 2022). In this study, we did not find a significant relationship between the serological results of the patients or seropositivity with the age and sex of the patients, and the presence of underlying diseases. However, upon comparing lymphocyte counts between the two groups, those whose SARS-CoV-2 IgG became positive during follow-up and those who remained negative, we observed a significant difference.

Our study encountered several limitations, notably regarding the acquisition of follow-up blood samples from all enrolled children. Some families expressed concerns about returning to the hospital due to fears of potential reinfection, leading to incomplete follow-up data. Additionally, the study was restricted by a relatively small sample size, potentially limiting the generalizability of the findings to a broader population. Furthermore, we did not assess the long-term persistence of antibodies, and the duration of immunity post-infection remains an area requiring further investigation.

## Conclusion

In conclusion, our study highlights the dynamic nature of SARS-CoV-2 IgG antibody responses in pediatric patients with COVID-19 infection. We observed a notable increase in seropositivity rates during follow-up. Furthermore, patients who were seropositive during follow-up demonstrated a more severe disease course and lower lymphocyte counts compared to those with persistently negative serology. Our findings underscore the importance of longitudinal serological monitoring in understanding disease progression and immune response dynamics in pediatric COVID-19 cases. Continuous follow-up of COVID-19 patients can provide valuable insights into their clinical conditions post-discharge, aiding in better patient management and healthcare decision-making.

## Data availability statement

The data that support the findings of this study are available from the corresponding authors upon reasonable request.

## Ethics statement

The studies involving humans were approved by the Tehran University of Medical Sciences. The studies were conducted in accordance with the local legislation and institutional requirements. Written informed consent for participation in this study was provided by the participants’ legal guardians/next of kin. Written informed consent was obtained from the individual(s), and minor(s)’ legal guardian/next of kin, for the publication of any potentially identifiable images or data included in this article.

## Author contributions

ShM: Conceptualization, Supervision, Writing – review and editing, Formal analysis, Methodology, Software, Validation, Visualization, Writing – original draft. BP: Investigation, Validation, Writing – review and editing, Conceptualization, Supervision. MAS: Investigation, Validation, Writing – review and editing, Formal analysis, Methodology. MS: Conceptualization, Methodology, Writing – review and editing, Investigation. EJ: Formal analysis, Visualization, Writing – original draft. RH: Investigation, Methodology, Writing – review and editing. SeM: Conceptualization, Funding acquisition, Investigation, Project administration, Supervision, Writing – review and editing.
